# Surgical anatomy of the recurrent laryngeal nerve: a systematic review and meta-analysis of variants

**DOI:** 10.1007/s00423-026-04097-0

**Published:** 2026-06-15

**Authors:** Properzi Luca, Vipul D. Yagnik, Matteucci Matteo, Filippo Sabatini, Bruno Cirillo, Antonio Pesce, Francesco Brucchi, Sara Luricella, Gianlorenzo Dionigi, Vito D’Adrea, Marco Artico, Flavio Forte, Roberto Cirocchi

**Affiliations:** 1 Dipartimento di Chirurgia Generale e Digestiva, Università degli Studi di Perugia, Ospedale “Santa Maria”, Terni, Terni Italy; 2 Department of Surgery, Banas Medical College and Research Institute, Palanpur, Gujarat India; 3 Department of Medicine and Surgery, University of Milan, Milan, Italy; 4 Department of Surgery, Sapienza University, Viale del Policlinico 155, Rome, 00161 Italy; 5 Department of Surgery, Azienda Unità Sanitaria Locale Ferrara, University of Ferrara, Lagosanto, FE Italy; 6 University of Milan, Milan, 20122 Italy; 7 Colorectal Surgery Division, Department of Surgery, Fondazione IRCCS Istituto Nazionale Dei Tumori, Milan, 20133 Italy; 8 Division of Surgery, Istituto Auxologico Italiano IRCCS (Istituto Di Ricovero E Cura a Carattere Scientifico), Via Giuseppe Mercalli, 30, Milan, 20122 Italy; 9 Department of Surgery, Sapienza University of Rome, Rome, Italy; 10 Department of SensoryOrgans, Sapienza University, Rome, 00161 Italy; 11 Urology Department of M.G, Vannini Hospital and Science Department of Roma Tre University, Rome, Italy

**Keywords:** Recurrent laryngeal nerve, Inferior thyroid artery, Non-recurrent laryngeal nerve

## Abstract

**Background:**

Injury to the recurrent laryngeal nerve (RLN) remains a significant concern in thyroid surgery. The inferior thyroid artery (ITA) is frequently used as a landmark. Yet, the RLN–ITA relationship shows substantial anatomical variability, and the non-recurrent laryngeal nerve (NRLN) represents a rare but high-risk variant. This systematic review and meta-analysis quantified the prevalence of RLN–ITA topographic patterns and NRLN occurrence.

**Materials and methods:**

A PRISMA-compliant search was performed across major databases up to May 1, 2025. Eligible studies included intraoperative, cadaveric, or mixed anatomical series reporting extractable numerical data on RLN–ITA relationships (anterior, posterior, interposed) and/or NRLN prevalence, using the hemisoma as the unit of analysis. Study quality was assessed with the AQUA tool. Proportions were pooled using Freeman–Tukey double arcsine transformation under a random-effects model with REML estimation. Heterogeneity was quantified with I², τ², and Cochran’s Q, and prespecified subgroup analyses were conducted by study setting.

**Results:**

Sixty studies (1973–2025) comprising 9,032 patients and 14,820 hemisomas were included. Fifty-seven studies contributed 14,169 hemisomas to the RLN–ITA synthesis. Crude proportions were posterior 54.43% (7,712/14,169), anterior 27.11% (3,841/14,169), and interposed 18.46% (2,616/14,169). Random-effects pooling confirmed a posterior course as the most frequent configuration (~ 52%, 95% CI ~ 46–58%), followed by anterior (~ 30%, 95% CI ~ 24–36%) and interposed (~ 18%, 95% CI ~ 15–21%), with very high heterogeneity (I² > 97%). Subgroup analyses of intraoperative (30 studies; 10,376 hemisomas) and cadaveric (25 studies; 3,589 hemisomas) series yielded similar distributions, with persistently high heterogeneity (I² > 94%). Across all studies, 39 NRLNs were identified, all on the right side, corresponding to a prevalence of 0.78% among right hemisomas (*n* = 4,974); no left-sided NRLNs were reported.

**Conclusion:**

The RLN most commonly courses posterior to the ITA, but anterior and interposed variants are sufficiently frequent to mandate systematic intraoperative verification before vessel ligation. The NRLN is rare and exclusively right-sided in available data, yet clinically significant due to its high injury risk. Marked between-study heterogeneity highlights the need for standardized definitions and side-specific reporting in future anatomical research.

## Introduction

Thyroid and parathyroid surgery requires accurate identification and preservation of the recurrent laryngeal nerve (RLN), as RLN injury may result in transient or permanent vocal fold dysfunction with significant functional and quality-of-life consequences. Although intraoperative nerve monitoring (IONM) has been adopted as an adjunct to visual nerve identification and standardized protocols have been proposed, safe dissection continues to rely primarily on a detailed understanding of RLN anatomy and its anatomical variability [1]. 

Recurrent laryngeal nerve (RLN) injury remains one of the most relevant complications of thyroidectomy. It may cause transient or permanent vocal fold dysfunction, leading to dysphonia, aspiration risk, and impaired quality of life, with potential need for rehabilitation or further interventions. Reported incidence varies across series depending on definitions, case-mix, and monitoring strategies; nevertheless, its clinical burden supports continued efforts to refine anatomical orientation and risk reduction [2]. 

Among the anatomical landmarks used to facilitate RLN localization, the inferior thyroid artery (ITA) and its branches are frequently referenced during thyroidectomy. However, the RLN–ITA relationship is highly variable: the nerve may course posterior to the artery, anterior to it, or between its branches, with reported prevalences differing substantially across studies, sides, and populations. Such variability limits the reliability of a single landmark-based strategy and contributes to inconsistent operative exposure and anatomical reporting [3–5]. 

A further high-risk anatomical variant is the non-recurrent laryngeal nerve (NRLN), which typically arises directly from the vagus nerve and is encountered almost exclusively on the right side, often in association with vascular anomalies such as an aberrant right subclavian artery. Although uncommon, failure to anticipate an NRLN may substantially increase the risk of iatrogenic nerve injury, highlighting the clinical importance of accurate, side-specific prevalence estimates and standardized anatomical reporting [6, 7]. 

Given the wide methodological and demographic variability across anatomical series, including both intraoperative observations and cadaveric dissections, a comprehensive synthesis is required to quantify RLN–ITA relationship patterns and NRLN prevalence using consistent denominators and explicit handling of non-identifiable or non-classifiable cases. The objective of this systematic review and random-effects meta-analysis aimed to pool the proportions of RLN courses relative to ITA branches (posterior, anterior, and interposed), estimate the prevalence of NRLN using an appropriate side-specific denominator, and explore heterogeneity according to study modality and other prespecified factors [3, 6]. Previous meta-analyses focus on the incidence of RLN injury and the prevalence of NRLN, but the quantitative synthesis of RLN-ITA topography and the prevalence of NRLN using side-specific denominators is limited, which justifies this meta-analysis.

## Materials and methods

### Search strategy and information sources

This systematic review was conducted in accordance with the PRISMA recommendations [8]. A comprehensive literature search was performed up to May 1, 2025 across the following databases: PubMed (MEDLINE), EMBASE, Web of Science, Scopus and Cochrane Central Register of Controlled Trials (CENTRAL). In addition, Google Scholar was searched, screening the first 200 results ranked by relevance. For each database, the search date and the number of records retrieved were documented. Records were deduplicated using dedicated reference management software (Rayyan); the deduplication criteria and the number of duplicates removed are reported in the PRISMA flow diagram. The protocol for this review was registered with PROSPERO with registration number CRD420251232692. The full protocol is available at https://www.crd.york.ac.uk/PROSPERO/view/CRD420251232692, and no amendments were made after registration.The search strategy combined controlled vocabulary terms (MeSH and Emtree, when available) with free-text keywords. An example of the PubMed search strategy, adapted to the syntax of the other databases, was as follows:

(“recurrent laryngeal nerve” OR “RLN” OR “laryngeal nerve, recurrent” OR

“non-recurrent laryngeal nerve” OR “NRLN”) AND

(“inferior thyroid artery” OR “inferior thyroid artery branches” OR

“thyroid artery”) AND

(“anatomical variation” OR “anatomic variation” OR “variation” OR

“variant” OR “relationship”)

Reference lists of all included studies were manually screened to identify additional relevant articles (reference chaining). No time limits or language restrictions were applied at the search stage; however, only studies with full text available in English or with an English translation were included. The complete search strategy and database-specific queries are provided in Appendix A.

### Eligibility criteria

Eligible studies were intraoperative, cadaveric, or mixed anatomical investigations reporting extractable numerical data on the topographic relationship between the recurrent laryngeal nerve (RLN) and branches of the inferior thyroid artery (ITA), classified as anterior, posterior, or interposed (between), and/or reporting the presence of a non-recurrent laryngeal nerve (NRLN). The unit of analysis was the hemisoma (right or left side).

Exclusion criteria included Review, case reports, studies with insufficient sample size (defined a priori as fewer than 10 hemisomas), letters, abstracts without full text, and studies lacking extractable numerical data relevant to the predefined outcomes.

### Study selection

Two reviewers independently screened titles and abstracts for eligibility. Full-text assessment was subsequently performed for potentially relevant studies. Disagreements at any stage of the selection process were resolved through discussion and consensus, and, when necessary, by consultation with a third reviewer.

### Data extraction and data handling

Data extraction was performed by two reviewers independently, using a predefined data collection form, with any discrepancies resolved through consensus. Each included study provided the following information extracted information : author, year of publication, country, type of anatomical assessment (intraoperative, cadaveric, or mixed), number of patients, total number of hemisomas analyzed, number of hemisomas classified according to the RLN–ITA relationship (anterior, posterior, or interposed), number of hemisomas in which the RLN was identified but not classifiable relative to the ITA, number of hemisomas in which the RLN was not identified, and number and side of non-recurrent laryngeal nerves.

Hemisomas reported as “identified but not specified” (or equivalent terminology) were recorded as identified but not classifiable and were excluded from the denominator used to pool the proportions of anterior, posterior, and interposed RLN–ITA relationships. These cases were retained in reconciliation tables and explicitly addressed as a limitation. Studies reporting partial data (e.g., right-sided hemisomas only) were included in side-specific analyses using the appropriate denominator. In cases where data were ambiguous or insufficient, attempts were made to reach out the original authors for clarification.

### Study quality assessment

Methodological quality and risk of bias of the included anatomical studies were assessed using the Anatomical Quality Assessment (AQUA) tool developed by the International Evidence-Based Anatomy Working Group. The AQUA tool comprises 20 items grouped into five domains: aim and subject characteristics, study design, characterization of methods (including techniques used to identify and dissect the recurrent laryngeal nerve and the inferior thyroid artery), descriptive anatomy (completeness and consistency of anatomical reporting, side-specific reporting, and handling of non-identifiable or non-classifiable cases), and results reporting (numerical reporting, transparency regarding missing data, study limitations, and conflicts of interest). Each domain was rated as low, unclear, or high risk of bias, and an overall risk of bias (low, moderate, or high) was assigned to each study based on the combination of the five domains. All 60 included studies underwent quality appraisal, which was also incorporated into sensitivity analyses to assess the impact of high-risk studies on pooled estimates.

### Statistical analysis

The primary outcomes were the proportion of hemisomas in which the RLN–ITA relationship was classified as posterior, anterior, or interposed (with the denominator restricted to hemisomas in which the relationship was classifiable) and the prevalence of non-recurrent laryngeal nerve (NRLN), calculated using the appropriate denominator (right-sided hemisomas analyzed). Proportions were pooled using the Freeman–Tukey double arcsine transformation, selected to stabilize variances for proportions close to 0 or 1 and for studies with small denominators, thereby reducing the risk of negative variance estimates compared with pooling untransformed proportions. Random-effects meta-analysis was performed, with between-study variance (τ²) estimated using restricted maximum likelihood (REML), which was preferred over the DerSimonian–Laird method in the presence of substantial heterogeneity and variable study sizes due to its lower bias in τ² estimation. Confidence intervals were calculated on the transformed scale and subsequently back-transformed to the proportion scale for reporting. In the Results section, raw counts and denominators were consistently reported alongside back-transformed estimates to ensure transparency. For all outcome effect measure was corresponding with 95% confidence interval. All meta-analyses were conducted in R (R Foundation for Statistical Computing, Vienna, Austria), using the meta and/or metafor packages (random-effects models with τ² estimated by REML; Freeman–Tukey double arcsine transformation for proportions).

### Heterogeneityassessment

For each outcome, heterogeneity was assessed using Cochran’s Q test (p value), I², τ² (with the estimation method specified), and the confidence interval for I². Interpretive thresholds for I² were defined as follows: 0–40% possibly unimportant heterogeneity, 30–60% moderate heterogeneity, 50–90% substantial heterogeneity, and 75–100% considerable heterogeneity. These thresholds were considered indicative, and final interpretation also incorporated clinical consistency and study precision. When I² exceeded 50%, τ² was emphasized to quantify the absolute magnitude of between-study variability. Prespecified exploratory analyses were conducted in the presence of substantial heterogeneity, including subgroup analyses by study type (intraoperative, cadaveric, or mixed), geographic area (continent), and decade of publication. When sufficient studies were available (minimum of 10 studies per covariate), meta-regression analyses were performed to explore potential sources of heterogeneity. A leave-one-out sensitivity analysis was also conducted to identify influential studies and assess the robustness of pooled estimates.

### Certainty of evidence

GRADE was not performed as it is not validated for descriptive anatomical study.

## Results

### Search strategy

**Fig. 1:**
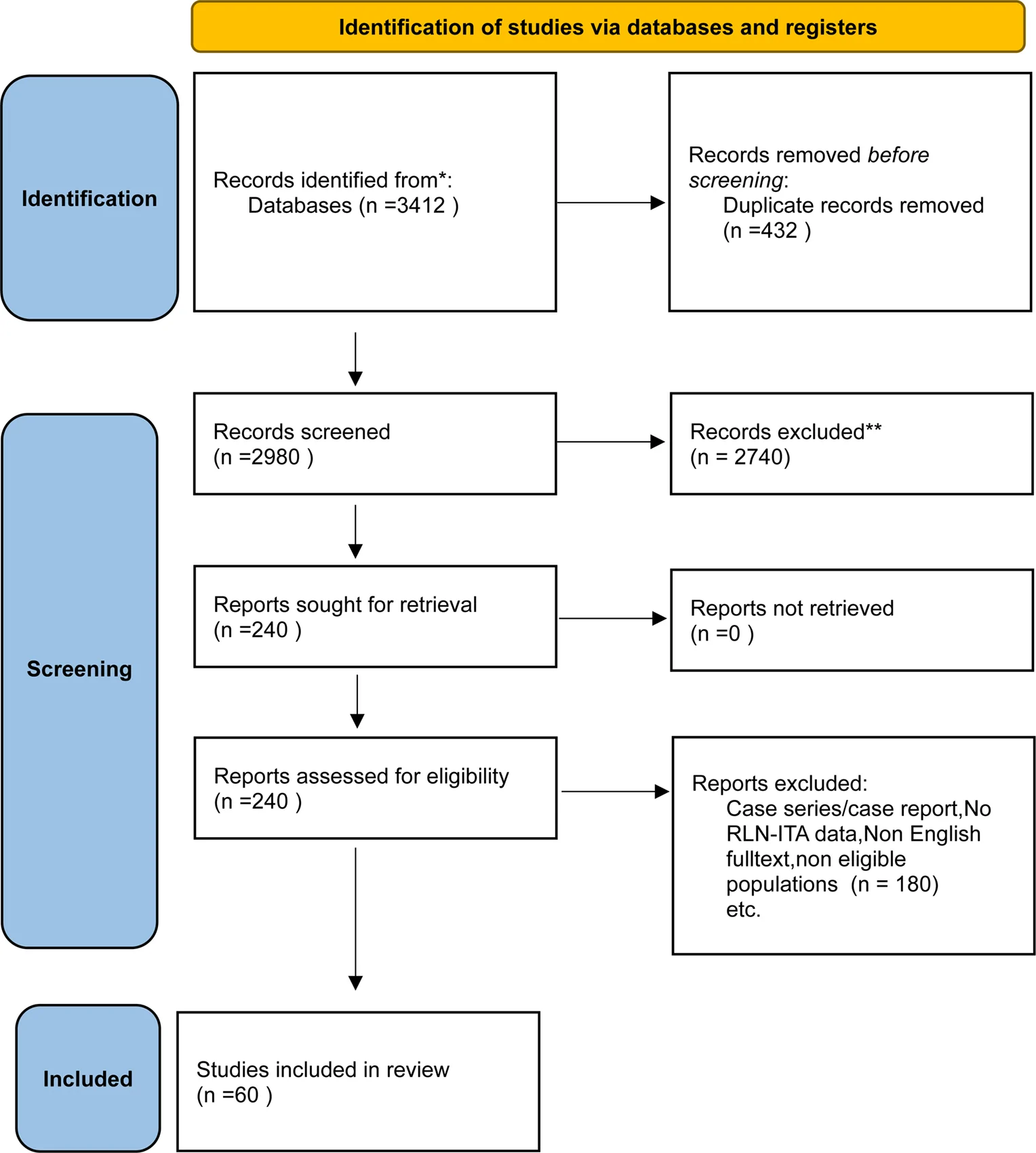
Prisma flowchart

The systematic search was conducted in accordance with PRISMA guidelines. A total of 3,412 records were identified across the selected databases. After removal of duplicates (*n* = 432), 2,980 records were screened based on titles and abstracts, of which 2,740 were excluded for irrelevance or failure to meet the inclusion criteria. Full-text assessment was subsequently performed for 240 articles; 180 studies were excluded after full-text review due to inappropriate study design (e.g., case reports or case series), absence of extractable data on anatomical variants, non-eligible study populations, or unavailability of the full text in English. Ultimately, 60 studies met the inclusion criteria and were included in the qualitative and quantitative synthesis (Fig. 1).

### Characteristics of the included studies

Overall, 60 studies were included in the systematic review. For the quantitative meta-analysis of the anatomical relationship between the recurrent laryngeal nerve (RLN) and the inferior thyroid artery (ITA), only studies reporting extractable numerical data for at least one of the three predefined variants (anterior, posterior, or interposed [between] course) were eligible. Accordingly, 57 studies contributed to the pooling of RLN–ITA proportions. Three studies (Gaurav 2023, Diouf 2023 [9], and Hemmaouil 2019 [10]) did not provide a numerical classification of RLN–ITA relationships and were therefore excluded from this specific meta-analysis, while remaining included in the overall qualitative synthesis.

The included studies were published between 1973 and 2025 and demonstrated broad geographic representation across Europe, the Americas, Asia, Africa, and Oceania. Collectively, they comprised a total of 9,032 patients and 14,820 hemisomas analyzed. Most studies were based on intraoperative assessments, a substantial proportion derived from cadaveric dissections, and a smaller number were mixed studies combining surgical and cadaveric observations.

For subgroup analyses of RLN–ITA relationships, the 57 studies providing quantitative data were stratified according to the type of anatomical assessment into intraoperative (surgical), cadaveric dissection, and mixed studies. Specifically, the meta-analysis included 30 intraoperative, 25 cadaveric, and 2 mixed studies, contributing a total of 14,169 classified hemisomas. This figure represents the global denominator used to pool the proportions of anterior, posterior, and interposed (between) RLN–ITA relationships. Thus, subgroup counts refer exclusively to the 57 studies reporting numerical RLN–ITA classifications, whereas the total number of studies included in the systematic review remained 60, as the three non-classifiable studies were retained for qualitative synthesis but excluded from this quantitative endpoint.

Table 1 summarizes, for each included study, the country of origin, type of anatomical assessment (intraoperative, cadaveric, or mixed), sample size, and the number of hemisomas classified for the RLN–ITA relationship analysis

**Table 1:** Characteristics of the included studies

Author-year of publication	Nation	Type of evaluation	Number of patientsincluded	Number of half- bodies
Nitsa 2025 [11]	Greece	Intraoperative study	60	116
Kiluba 2024 [12]	South Africa + Arab Emirates	Mixed: Intraoperative study	79	98
Dass 2024 [13]	India	Cadavericdissection	64	128
Yilmaz 2024 [14]	Turkey	Intraoperative study	91	150
Torres Morientes 2024 [15]	Spain	Intraoperative study	440	695
Qaiser 2024 [16]	Pakistan	Intraoperative study	64	128
Pant 2024 [17]	India	Intraoperative study	15	15
Kiluba 2024 [12]b	South Africa + Arab Emirates	Mixed: Cadavericdissection	50	98
Dadwani 2024 [18]	India	Cadavericdissection	40	80
Gurluler 2024 [19]	Turkey	Intraoperative study	421	842
Aygun 2024 [20]	Turkey	Intraoperative study	663	1070
Erfan 2024 [21]	India	Intraoperative study	50	50
Dundar 2024 [22]	USA	Cadavericdissection	32	64
Abeysurya 2023 [23]	Sri Lanka	Cadavericdissection	35	70
Gkrinia 2023 [24]	Greece	Intraoperative study	64	128
Gaurav 2023 [25]	India on Africantribals	Intraoperative study	100	120
Samorekar 2023 [26]	India	Intraoperative study	50	100
Apoorva 2023 [27]	India	Intraoperative study	54	64
Kumar 2023 [28]	India	Intraoperative study	52	56
Patra 2023 [29]	India	Cadavericdissection	30	60
Diouf 2023 [9]	Senegal	Intraoperative study	30	53
Kiluba 2022 [30]	South Africa	Cadavericdissection	31	61
Anand 2022 [31]	India	Cadavericdissection	40	80
Kastan 2022 [32]	Turkey	Cadavericdissection	42	84
Kostek 2021 [33]	Turkey	Intraoperative study	696	1117
Gujrathi 2021 [34]	India	Intraoperative study	35	48
Islam 2020 [35]	Bangladesh	Mixed: Cadavericdissection	64	106
Thomas 2020 [36]	USA	Cadavericdissection	55	111
Niranjan 2020 [37]	Sri Lanka	Cadavericdissection	156	312
Hemmaouil 2019 [10]	Marocco	Intraoperative study	60	99
Saldanha 2019 [38]	India	Intraoperative study	91	150
Sreejayan 2019 [39]	India	Intraoperative study	393	735
Sheikh 2019 [40]	Pakistan	Intraoperative study	51	104
Shamkuwar 2016 [41]	India	Cadavericdissection	60	120
Saknure 2016 [42]	India	Cadavericdissection	100	200
Wojtczak 2018 [43]	Poland	Intraoperative study	128	243
Nyeki 2015 [44]	Cameroon	Intraoperative study	56	62
Zada 2014 [45]	Pakistan	Intraoperative study	271	416
Idris 2013 [46]	Sudan	Intraoperative study	82	164
Pradeep 2012 [47]	India	Intraoperative study	404	584
Ashgharpour 2012 [48]	UK	Cadavericdissection	143	246
Tang 2012 [49]	China	Cadavericdissection	80	160
Kaisha 2011 [50]	Kenya	Cadavericdissection	73	146
Sunanda 2010 [51]	Sri Lanka	Intraoperative study	26	43
Lee 2009 [52]	Korea	Cadavericdissection	55	110
Makay 2008 [53]	Turkey	Intraoperative study	253	487
Uen 2006 [54]	Taiwan	Cadavericdissection	60	120
Ardito 2004 [55]	Italy	Intraoperative study	1543	1856
Page 2003 [56]	France	Intraoperative study	251	481
Sun 2001 [57]	China	Cadavericdissection	50	100
Poyraz and Calguner 2001 [58]	Turkey	Cadavericdissection	30	48
Campos and Henrique 2000 [59]	Brazil	Cadavericdissection	76	142
Sturniolo et al. 1999 [60]	Italy	Intraoperative study	192	384
Salama and McGrath 1992 [61]	Australia	Cadavericdissection	138	144
Lekacos et al. 1992 [62]	Greece	Intraoperative study	172	191
Al-Salihi and Dabbagh 1989 [63]	Iraq	Cadavericdissection	106	212
Steinberg et al. 1986 [64]	South Africa	Cadavericdissection	90	180
Papadatos 1978 [65]	Suisse	Cadavericdissection	239	478
Skandalakis et al. 1976 [66]	Georgia	Cadavericdissection	102	203
Kratz 1973 [67]	Newpor, Ky	Intraoperative study	54	108

In a total of 249 hemisomas, the recurrent laryngeal nerve (RLN) was not identified. This finding reflects both methodological variability among studies and inherent challenges related to intraoperative visualization or documentation during anatomical dissections. Study-specific counts of non-identified RLNs are detailed in Table 2.

**Table 2:** Reports, for each study, the number of non-identified recurrent laryngeal nerves (RLNs)

Author-year of publication	Recurrent laryngeal nerves not indentified
Nitsa 2025 [11]	2
Kiluba 2024 [12]	22
Dass 2024 [13]	3
Yilmaz 2024 [14]	0
Torres Morientes 2024 [15]	5
Qaiser 2024 [16]	0
Pant 2024 [17]	0
Kiluba 2024 [12]b	0
Dadwani 2024 [18]	7
Gurluler 2024 [19]	0
Aygun 2024 [20]	4
Erfan 2024 [21]	0
Dundar 2024 [22]	0
Abeysurya 2023 [23]	3
Gkrinia 2023 [24]	0
Gaurav 2023 [25]	15
Samorekar 2023 [26]	31
Apoorva 2023 [27]	0
Kumar 2023 [28]	0
Patra 2023 [29]	0
Diouf 2023 [9]	0
Kiluba 2022 [30]	1
Anand 2022 [31]	0
Kastan 2022 [32]	0
Kostek 2021 [33]	0
Gujrathi 2021 [34]	0
Islam 2020 [35]	0
Thomas 2020 [36]	0
Niranjan 2020 [37]	0
Hemmaouil 2019 [10]	0
Saldanha 2019 [38]	10
Sreejayan 2019 [39]	2
Sheikh 2019 [40]	22
Wojtczak 2018 [43]	0
Saknure 2016 [42]	0
Shamkuwar 2016 [41]	0
Nyeki 2015 [44]	0
Zada 2014 [45]	18
Idris 2013 [46]	0
Pradeep 2012 [47]	0
Ashgharpour 2012 [48]	0
Tang 2012 [49]	0
Kaisha 2011 [50]	0
Sunanda 2010 [51]	0
Lee 2009 [52]	0
Makay 2008 [53]	0
Uen 2006 [54]	0
Ardito 2004 [55]	0
Page 2003 [56]	0
Sun 2001 [57]	0
Poyraz and Calguner 2001 [58]	0
Campos and Henrique 2000 [59]	0
Sturniolo et al. 1999 [60]	104
Salama and McGrath 1992 [61]	0
Lekacos et al. 1992 [62]	0
Al-Salihi and Dabbagh 1989 [63]	0
Steinberg et al. 1986 [64]	0
Papadatos 1978 [65]	0
Skandalakis et al. 1976 [66]	0
Kratz 1973 [67]	0

### Methodological quality assessment (AQUA)

Application of the Anatomical Quality Assessment (AQUA) tool to the 60 included studies indicated an overall moderate methodological quality. In the Aim and subject characteristics and Characterization of methods domains—particularly among more recent studies—the risk of bias was predominantly low, supported by clearly stated objectives focused on RLN–ITA variants and relatively detailed descriptions of intraoperative or cadaveric dissection techniques.

In contrast, the Study design and Results reporting domains frequently received unclear or high risk-of-bias ratings, especially in older studies, in which sampling methods were poorly described (e.g., non-consecutive case series or unreported inclusion criteria) and results were incompletely reported. Notably, side-specific reporting of the recurrent laryngeal nerve was not systematic across all studies, and a non-negligible proportion of hemisomas were described as “identified” without a precise classification of their relationship with the inferior thyroid artery.

Within the Descriptive anatomy domain, most studies provided at least a basic categorization of the RLN–ITA relationship as anterior, posterior, or interposed (“between”). However, inconsistencies persisted in the handling and reporting of non-identified nerves and non-classifiable hemisomas, contributing to information bias. This observation is consistent with the aggregated counts of 651 hemisomas identified but not classifiable with respect to the ITA and 249 RLNs reported as not identified, which together represent a major source of uncertainty in the pooled estimates.

Considering all five AQUA domains, only a minority of studies were judged to be at low overall risk of bias, whereas most were classified as moderate and a smaller number as high risk of bias. High-risk studies were typically characterized by very small sample sizes, fragmented methodological descriptions, or incomplete reporting of RLN–ITA relationships. These methodological limitations were taken into account when interpreting the meta-analytical results and discussing the findings.

### Analysis of RLN–ITA relationships

In a total of 14,169 hemisomas, the anatomical relationship between the recurrent laryngeal nerve (RLN) and branches of the inferior thyroid artery (ITA) was classified (Table 3). When comparing the total number of hemisomas reported across all included studies (Table 1: 14,820) with the number of hemisomasactually classified according to their RLN–ITA relationship (Table 3: 14,169), a discrepancy of 651 hemisomas was observed. This discrepancy reflects studies in which the RLN was reported as identified but the topographic relationship with the ITA was not specified.

**Table 3:** summarizes, for each included study, the reported anatomical relationships between the recurrent laryngeal nerve (RLN) and the inferior thyroid artery (ITA). Nd/NR indicates data not reported or excluded from pooling for this specific endpoint

Author-Year of publication	RLN TYPE		
	Anterior	Posterior	Between
Nitsa 2025 [11]	15	65	36
Kiluba 2024 [12]	33	29	36
Dass 2024 [13]	29	33	8
Yilmaz 2024 [14]	29	104	17
Torres Morientes 2024 [15]	214	286	184
Qaiser 2024 [16]	4	124	0
Pant 2024 [17]	10	5	0
Kiluba 2024 [12]	33	29	36
Dadwani 2024 [18]	18	51	4
Gurluler 2024 [19]	162	600	80
Aygun 2024 [20]	487	473	102
Erfan 2024 [21]	14	36	0
Dundar 2024 [22]	4	51	9
Abeysurya 2023 [23]	35	24	8
Gkrinia 2023 [24]	35	Nd	Nd
Gaurav 2023 [25]	Nd	Nd	Nd
Samorekar 2023 [26]	23	43	3
Apoorva 2023 [27]	17	25	12
Kumar 2023 [28]	10	46	0
Patra 2023 [29]	18	39	3
Diouf 2023 [9]	Nd	Nd	Nd
Anand 2022 [31]	2	69	9
Kastan 2022 [32]	0	77	7
Kiluba 2022 [30]	38	5	18
Kostek 2021 [33]	511	500	106
Gujrathi 2021 [34]	16	27	5
Islam 2020 [35]	19	62	25
Thomas 2020 [36]	53	53	5
Niranjan 2020 [37]	45	198	69
Saldanha 2019 [38]	125	15	0
Sreejayan 2019 [39]	52	476	205
Sheikh 2019 [40]	41	20	21
Hemmaouil 2019 [10]	Nd	Nd	Nd
Wojtczak 2018 [43]	53	186	4
Shamkuwar 2016 [41]	44	68	6
Saknure 2016 [42]	46	94	60
Nyeki 2015 [44]	13	40	9
Zada 2014 [45]	220	138	40
Idris 2013 [46]	61	63	40
Pradeep 2012 [47]	161	375	48
Ashgharpour 2012 [48]	81	88	77
Tang 2012 [49]	66	77	17
Kaisha 2011 [50]	54	75	17
Sunanda 2010 [51]	4	28	11
Lee 2009 [52]	35	50	25
Makay 2008 [53]	111	344	32
Uen 2006 [54]	17	79	24
Ardito 2004 [55]	131	1282	443
Page 2003 [56]	159	244	78
Sun 2001 [57]	32	39	29
Poyraz and Calguner 2001 [58]	8	18	22
Campos and Henrique 2000 [59]	40	35	67
Sturniolo et al. 1999 [60]	87	121	72
Salama and McGrath 1992 [61]	28	64	52
Lekacos et al. 1992 [62]	31	97	63
Al-Salihi and Dabbagh 1989 [63]	49	114	49
Steinberg et al. 1986 [64]	58	110	12
Papadatos 1978 [65]	96	191	191
Skandalakis et al. 1976 [66]	42	85	76
Kratz 1973 [67]	22	42	44

The 651 non-classified hemisomas originated from the following studies: Dass 2024 [13] (58 cases), Torres Morientes 2024 [15], Indian Journal 2024, Aygun 2024, Abeysurya 2023 [23], Gkrinia 2023 [24], Gaurav 2023 [25], Samorekar 2023, Apoorva 2023, Diouf 2023 [9], Hemmaouil 2019 [10], Saldanha 2019 [38], Sreejayan 2019, Sheikh 2019 [40], Shamkuwar 2016 [41], Zada 2014 [45], and Sturniolo et al. 1999. The sum of these values equals 651, confirming the source of the discrepancy.

An additional issue concerns the 249 non-identified RLNs, reported separately in Table 2. Detailed cross-checking revealed that 224 of these cases were already included within the 651 non-classified hemisomas, as the original studies reported them as “not identified” or “identified but not classified” without a clear methodological distinction. Consequently, only a small subset of 25 cases represents truly additional observations not encompassed by the 651 non-classified hemisomas, originating from Nitsa 2025 [11], Kiluba 2024 [12], and Kiluba 2022 [30].

The crude distributions of RLN–ITA relationships are summarized in Table 3. A posterior course of the recurrent laryngeal nerve was observed in 7,712 of 14,169 hemisomas (54.43%), whereas an anterior course was identified in 3,841 hemisomas (27.11%) and an interposed (“between”) course in 2,616 hemisomas (18.46%).

**Fig. 2:**
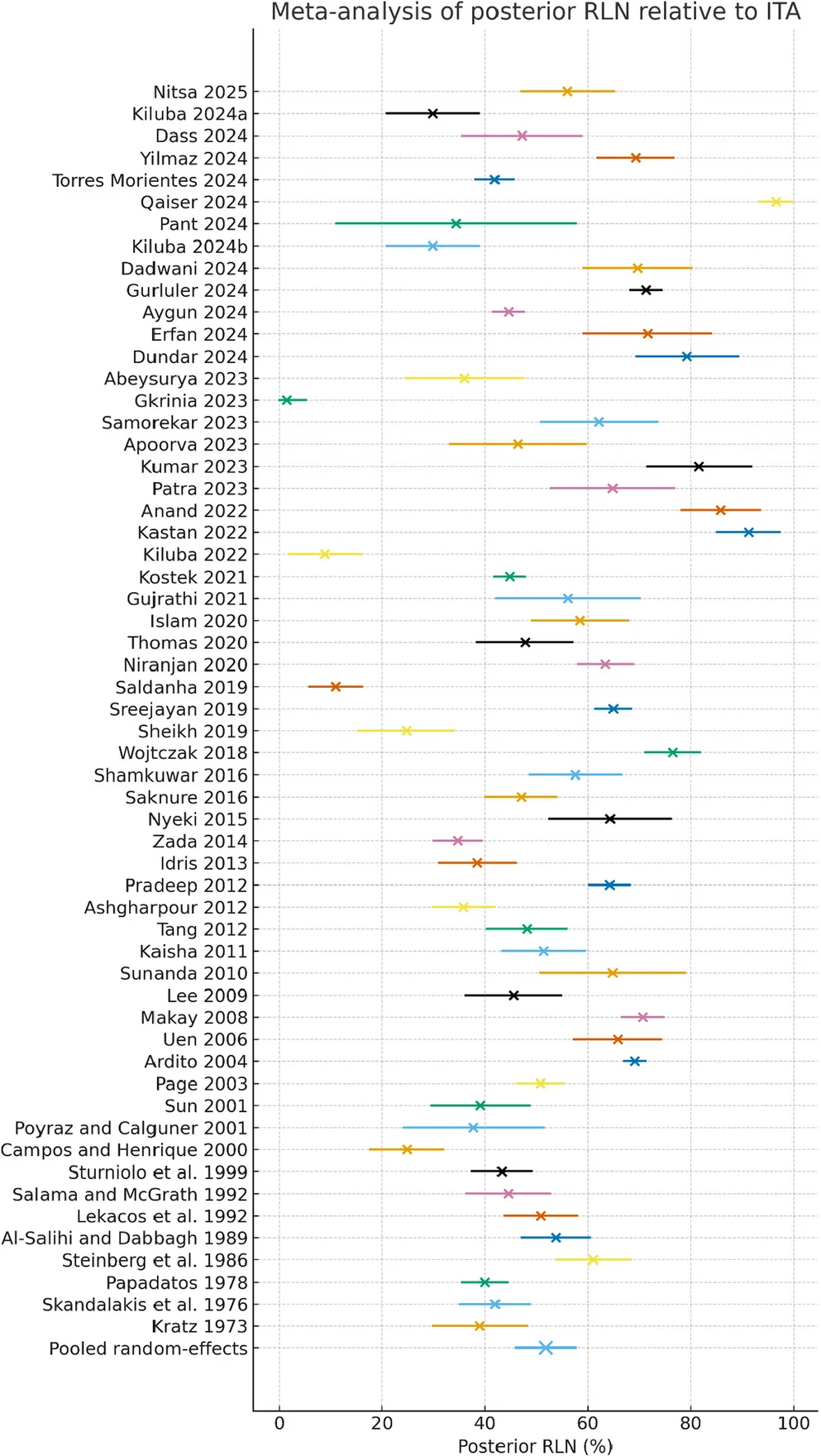
Posterior RLN relative to the ITA

**Fig. 3:**
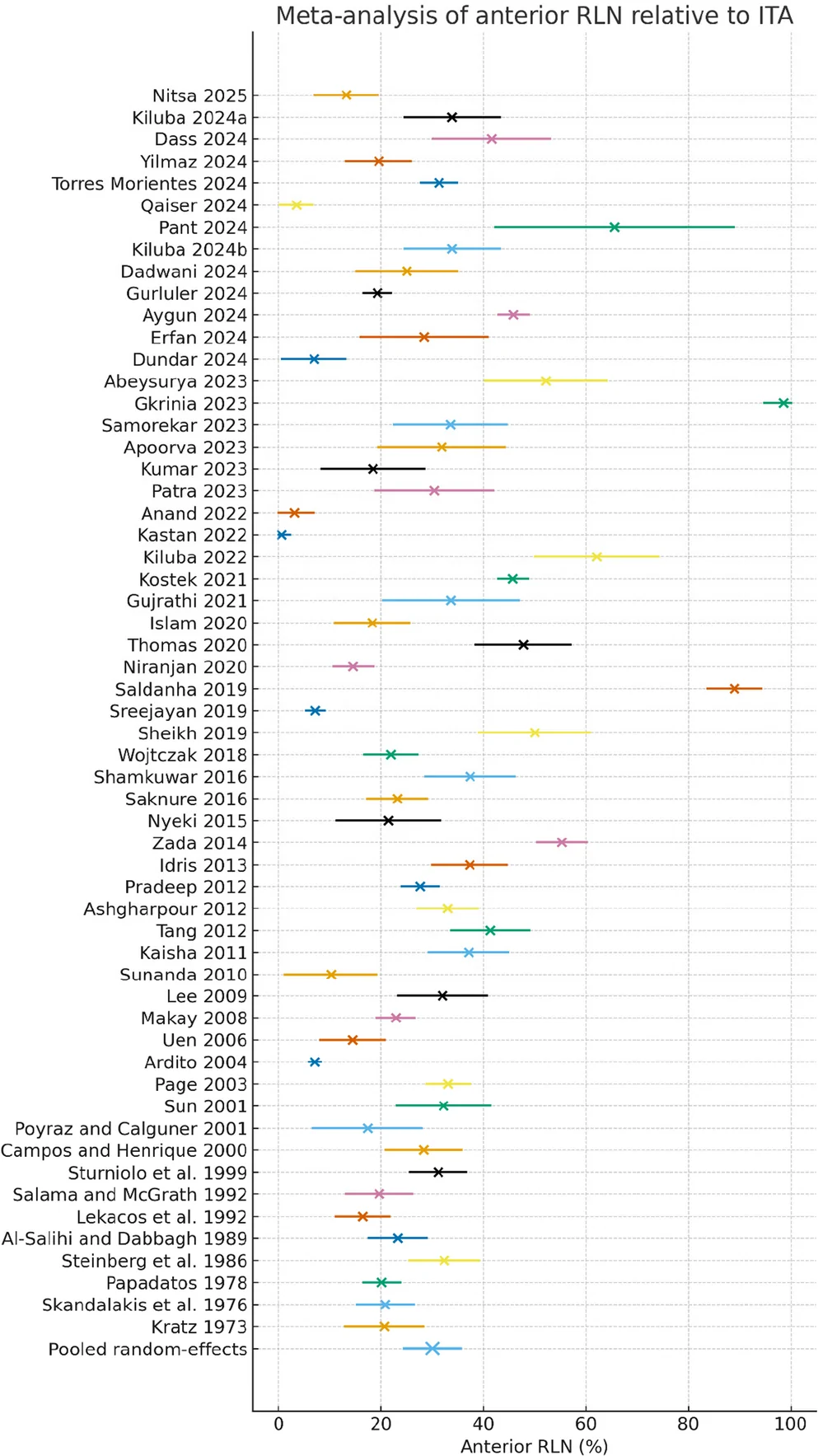
Anterior RLN relative to the inferior thyroid artery (ITA)

**Fig. 4:**
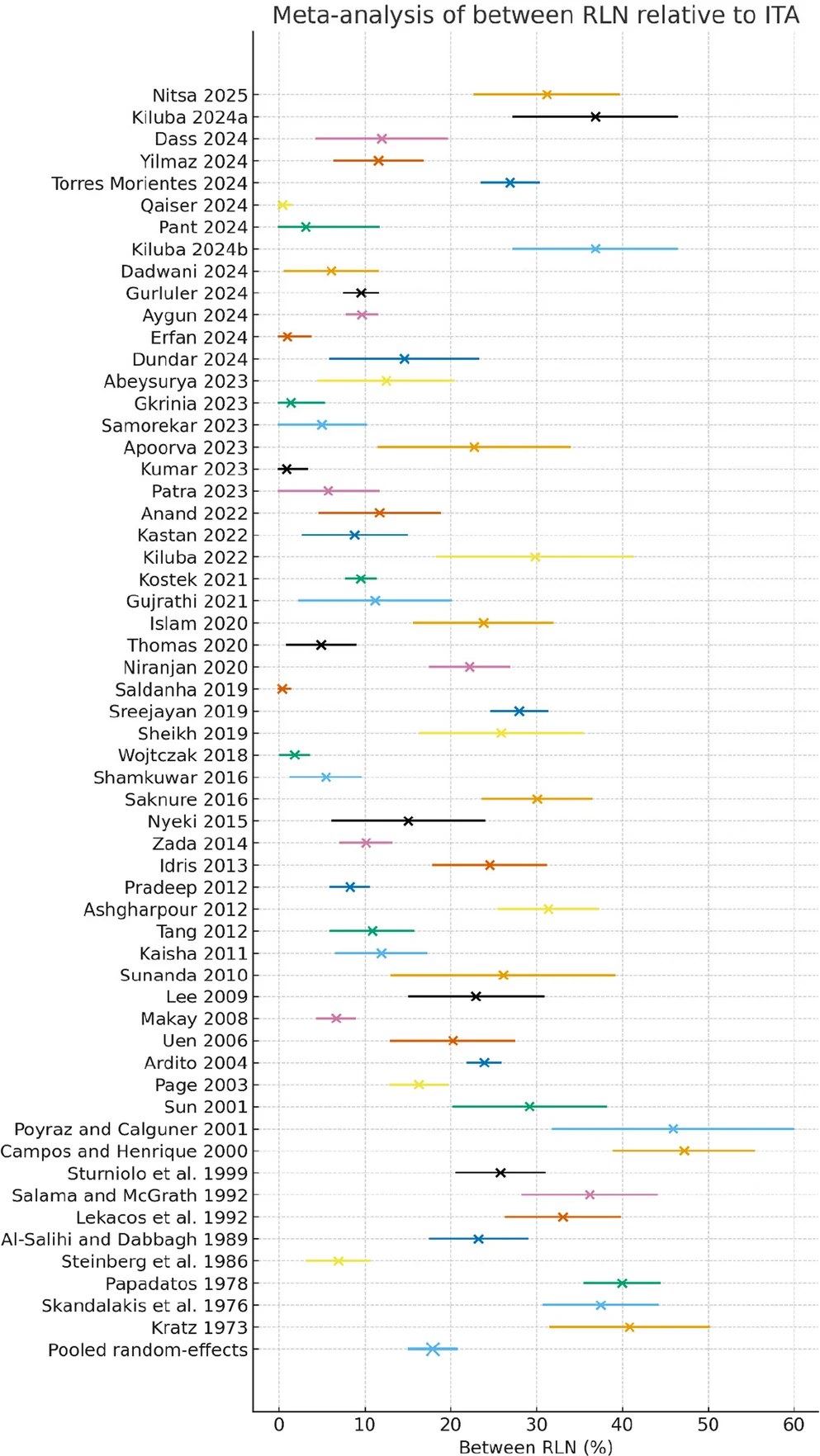
RLN between the branches of the inferior thyroid artery (ITA)

Random-effects meta-analysis confirmed that a posterior relationship between the RLN and ITA branches was the most frequent anatomical pattern, with a pooled proportion of approximately 52% (95% CI 46–58%; Fig. 2). The anterior and interposed variants were less common, with pooled estimates of approximately 30% (95% CI 24–36%; Fig. 3) and 18% (95% CI 15–21%; Fig. 4), respectively.

Heterogeneity was considerable for all anatomical categories (I² > 97%), reflecting substantial variability across studies in terms of anatomical assessment setting (intraoperative versus cadaveric), study populations, and methodological characteristics.

**Fig. 5:**
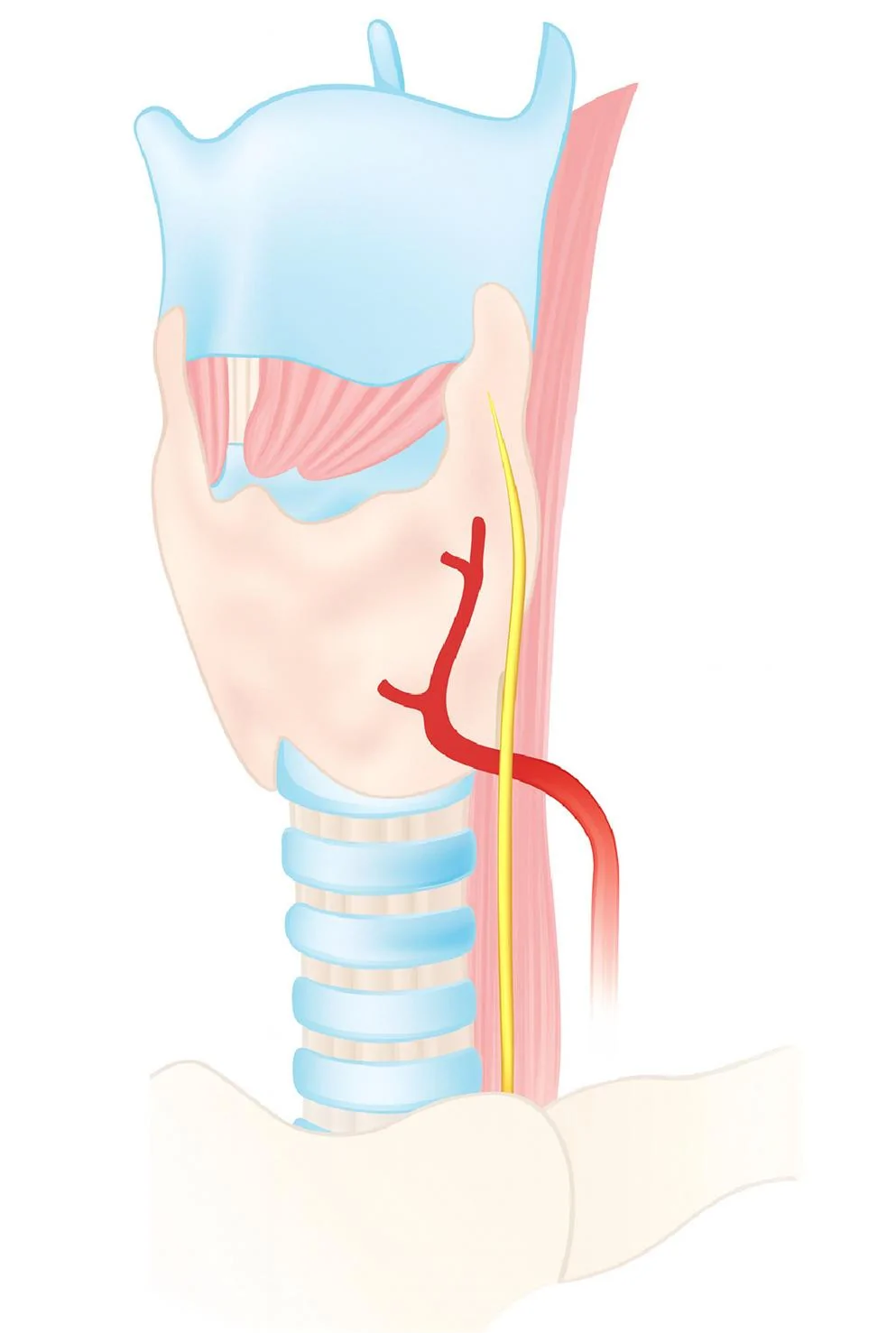
Relationships between RLN and ITA: RLN anterior to the ITA

**Fig. 6:**
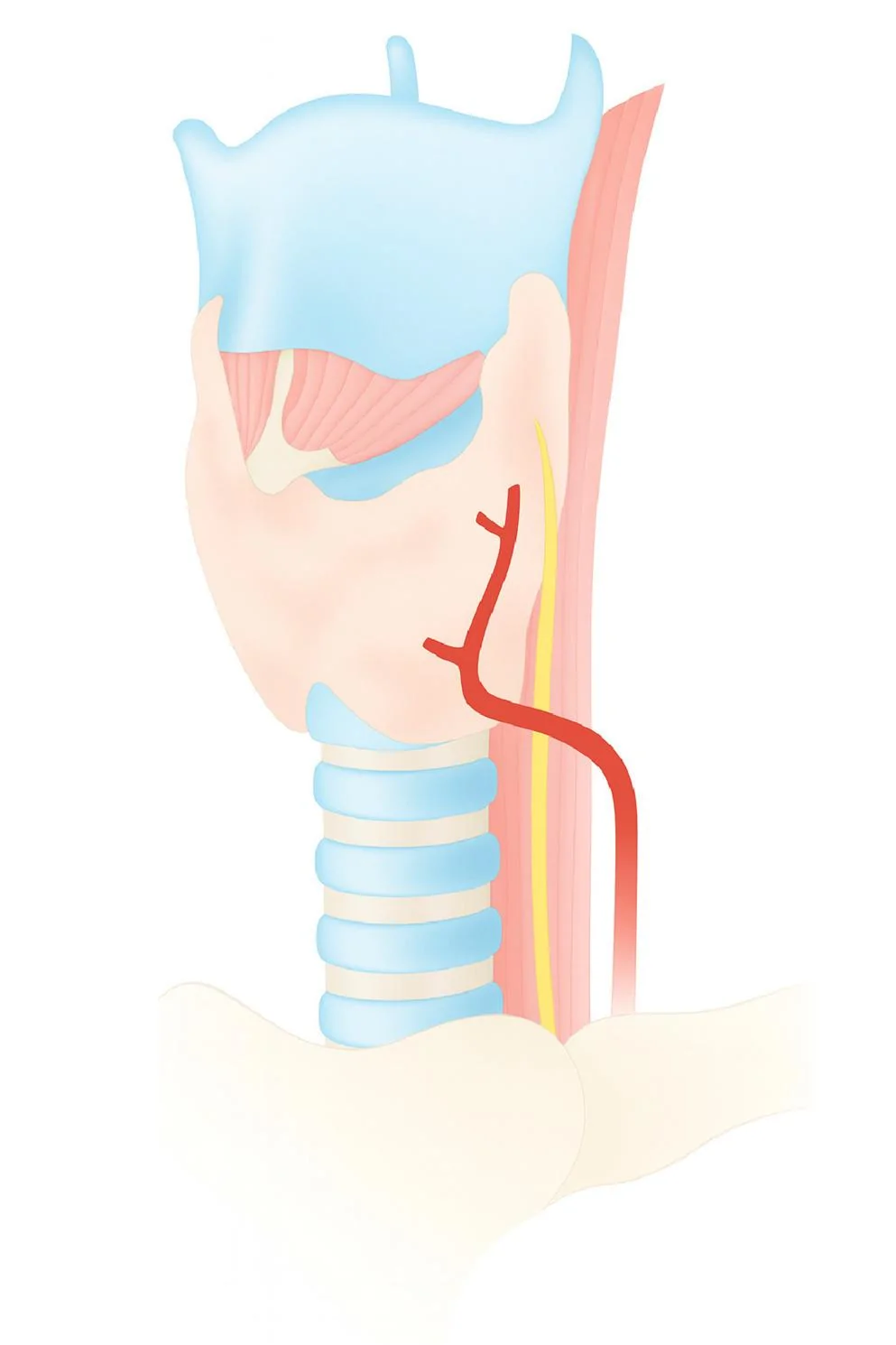
Relationships between RLN and ITA. RLN posterior to the ITA

**Fig. 7:**
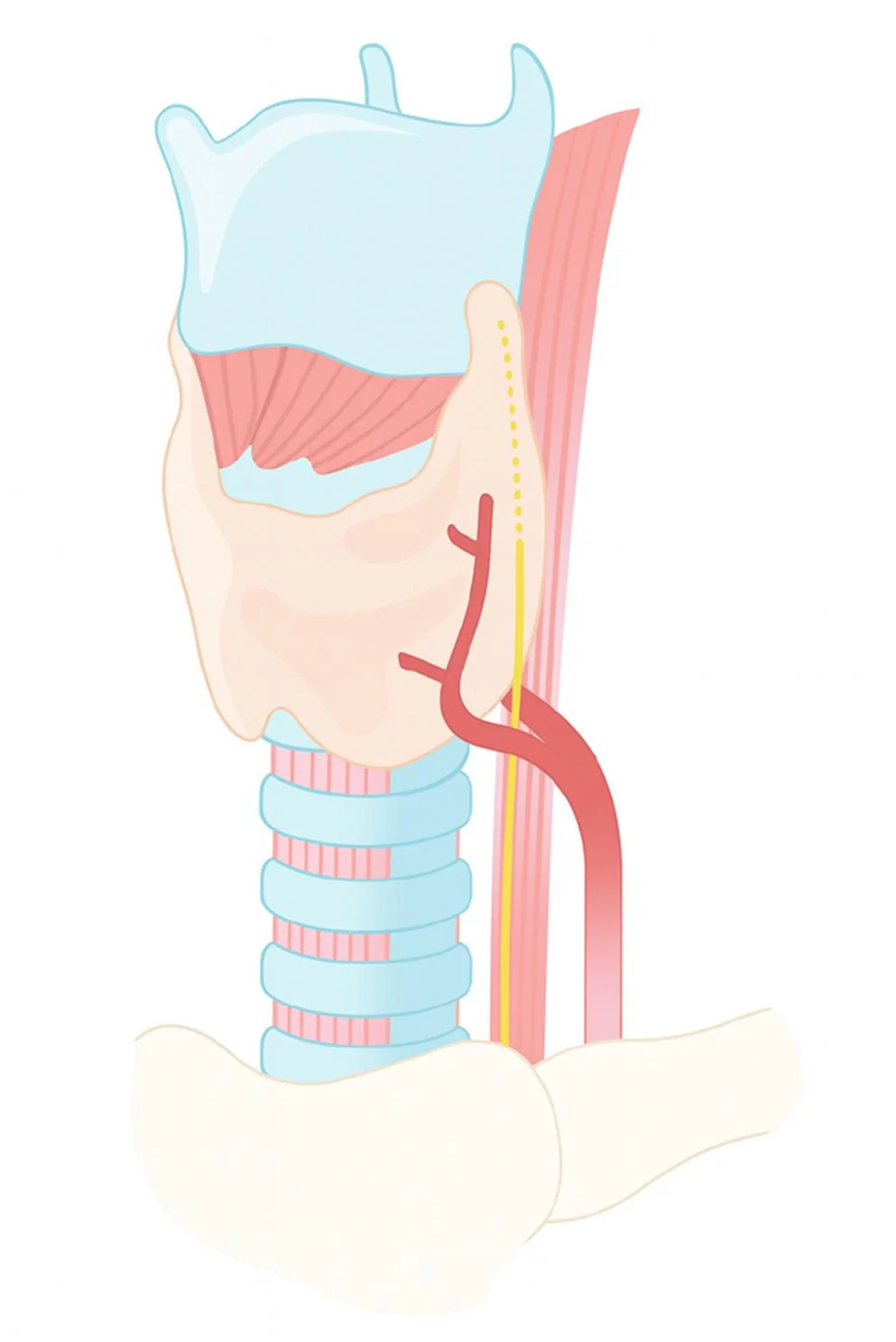
Relationships between RLN and ITA.: RLN between the branches of the ITA

Random-effects meta-analysis of the included studies demonstrated that a posterior course of the RLN relative to the ITA branches was the most common anatomical variant, with a pooled proportion of approximately 52% (95% CI 46–58%). The anterior and interposed (“between”) variants were less frequent, with pooled estimates of approximately 30% (95% CI 24–36%) and 18% (95% CI 15–21%), respectively. Heterogeneity was considerable across all anatomical categories (I² > 97%), likely reflecting differences in study setting (intraoperative versus cadaveric), study populations, and methodological characteristics among the included series (Figs. 2, 3, 4, 5, 6 and 7).

In subgroup analyses, intraoperative studies (30 studies; 10,376 hemisomas) demonstrated a posterior course of the recurrent laryngeal nerve relative to the inferior thyroid artery branches in approximately 53% of cases (95% CI 44–61%), with an anterior course observed in about 32% (95% CI 24–41%) and an interposed (“between”) course in approximately 15% (95% CI 12–18%). Cadaveric dissection studies (25 studies; 3,589 hemisomas) showed a comparable overall pattern, with a posterior course in approximately 51% of hemisomas (95% CI 42–59%), a slightly lower proportion of anterior variants (approximately 27%, 95% CI 21–34%), and a modestly higher proportion of interposed variants (approximately 21%, 95% CI 16–26%).

Heterogeneity remained considerable in both subgroups (I² > 94%), indicating substantial methodological, anatomical, and demographic variability across the included series.

Side-specific analyses (Table 4) further suggested a higher frequency of a posterior RLN course on the left side compared with the right, with differences varying according to study characteristics and assessment setting. Table 4 summarizes, for each study, the author, year of publication, and the distribution of RLN variants on the right and left sides of the neck. Left–right comparisons in Table 4 are presented descriptively, as bilateral sides within individuals were not consistently reported across studies and within-subject clustering could not be modeled.

**Table 4:** Distribution of recurrent laryngeal nerve (RLN) anatomical variants according to the right and left sides of the neck for each included study, with corresponding author and year of publication. *Nd/NR* indicates data not reported or excluded from pooling for this specific endpoint

Author- Year of publication	Right RLN			Left RLN		
	Typeanterior	Typeposterior	Typebetween	Typeanterior	Typeposterior	Typebetween
Nitsa 2025 [11]	Nd	Nd	Nd	Nd	Nd	Nd
Kiluba 2024 [12]	12	24	13	21	5	23
Dass 2024 [13]	13	19	6	16	14	2
Yilmaz 2024 [14]	17	47	10	12	57	7
Torres Morientes 2024 [15]	185	58	112	29	228	72
Qaiser 2024 [16]	1	63	0	3	61	0
Pant 2024 [17]	Nd	Nd	Nd	Nd	Nd	Nd
Kiluba 2024 [12]b	12	24	13	21	5	23
Dadwani 2024 [18]	Nd	Nd	Nd	Nd	Nd	Nd
Gurluler 2024 [19]	89	288	44	73	312	36
Aygun 2024 [20]	287	189	64	200	284	38
Erfan 2024 [21]	8	26	0	6	10	0
Dundar 2024 [22]	4	20	8	1	31	0
Abeysurya 2023 [23]	17	10	5	18	14	3
Gkrinia 2023 [24]	Nd	Nd	Nd	Nd	Nd	Nd
Gaurav 2023 [25]	Nd	Nd	Nd	Nd	Nd	Nd
Samorekar 2023 [26]	14	22	1	9	21	2
Apoorva 2023 [27]	20	30	14	8	46	10
Kumar 2023 [28]	6	29	0	4	17	0
Patra 2023 [29]	Nd	Nd	Nd	Nd	Nd	Nd 0
Diouf 2023 [9]	Nd	Nd	Nd	Nd	Nd	Nd
Anand 2022 [31]	2	36	2	0	33	7
Kastan 2022 [32]	Nd	Nd	Nd	Nd	Nd	Nd
Kiluba 2022 [30]	20	6	4	18	12	1
Kostek 2021 [33]	299	200	68	212	300	38
Gujrathi 2021 [34]	6	17	3	10	10	2
Thomas 2020 [36]	36	17	2	17	36	3
Islam 2020 [35]	15	25	15	4	37	10
Niranjan 2020 [37]	28	79	48	17	119	21
Hemmaouil 2019 [10]	36	0	16	0	41	7
Saldanha 2019 [38]	74	4	0	51	11	0
Sreejayan 2019 [39]	Nd	Nd	Nd	Nd	Nd	Nd
Sheikh 2019 [40]	17	7	18	24	13	3
Wojtczak 2018 [43]	23	92	4	30	94	0
Shamkuwar 2016 [41]	33	22	3	11	46	3
Saknure 2016 [42]	29	22	49	17	72	11
Nyeki 2015 [44]	10	17	5	3	23	4
Zada 2014 [45]	115	66	26	105	72	14
Idris 2013 [46]	Nd	Nd	Nd	Nd	Nd	Nd
Pradeep 2012 [47]	Nd	Nd	Nd	Nd	Nd	Nd
Ashgharpour 2012 [48]	Nd	Nd	Nd	Nd	Nd	Nd
Tang 2012 [49]	60	8	12	6	69	5
Kaisha 2011 [50]	Nd	Nd	Nd	Nd	Nd	Nd
Sunanda 2010 [51]	Nd	Nd	Nd	Nd	Nd	Nd
Lee 2009 [52]	22	19	14	13	31	11
Makay 2008 [53]	61	163	19	50	181	13
Uen 2006 [54]	12	37	11	5	42	13
Ardito 2004 [55]	113	576	256	18	706	187
Page 2003 [56]	137	68	48	22	176	30
Sun 2001 [57]	25	11	14	7	28	15
Poyraz and Calguner 2001 [58]	7	7	9	1	11	13
Campos and Henrique 2000 [59]	27	8	35	13	27	32
Sturniolo et al. 1999 [60]	44	56	41	43	65	31
Salama and McGrath 1992 [61]	14	29	29	14	35	23
Lekacos et al. 1992 [62]	38	52	19	12	45	25
Al-Salihi and Dabbagh 1989 [63]	36	31	39	13	83	10
Steinberg et al. 1986 [64]	Nd	Nd	Nd	Nd	Nd	Nd
Papadatos 1978 [65]	Nd	Nd	Nd	Nd	Nd	Nd
Skandalakis et al. 1976 [66]	Nd	Nd	Nd	Nd	Nd	Nd
Kratz 1973 [67]	14	16	26	8	26	18

### Analysis of non-recurrent laryngeal nerves (NRLN)

Across the 60 included studies, 39 non-recurrent laryngeal nerves (NRLNs) were reported (Table 5). Because this anatomical variant occurs almost exclusively on the right side, its frequency was calculated using the total number of right-sided hemisomas (*n* = 4,974); no left-sided cases were observed. This finding is consistent with embryological evidence and the well-established association between NRLN and right-sided occurrence.

Most NRLNs were reported in intraoperative series, with fewer cases identified in cadaveric dissections, suggesting that detection of this rare variant may be facilitated in surgical settings due to greater nerve exposure. The overall prevalence, referenced to right-sided hemisomas, was 0.78%, confirming that NRLN is uncommon but clinically relevant, particularly in thyroid surgery.

**Table 5:** Number and side of non-recurrent laryngeal nerves (NRLNs) identified in each included study. *Nd/NR* indicates data not reported or excluded from pooling for this specific endpoint

Author- Year of publication	NRLN	
	Nr.	Right/left
Nitsa 2025 [11]	0	0
Kiluba 2024 [12]	0	0
Dass 2024 [13]	NR	NR
Yilmaz 2024 [14]	1	Right
Torres Morientes 2024 [15]	6	Right
Qaiser 2024 [16]	NR	NR
Pant 2024 [17]	0	0
Kiluba 2024 [12]	1	Nr
Dadwani 2024 [18]	0	0
Gurluler 2024 [19]	0	0
Aygun 2024 [20]	4	Right
Erfan 2024 [21]	0	0
Dundar 2024 [22]	0	0
Abeysurya 2023 [23]	0	0
Gkrinia 2023 [24]	0	0
Gaurav 2023 [25]	1	Right
Samorekar 2023 [26]	0	0
Apoorva 2023 [27]	1	Right
Kumar 2023 [28]	0	0
Diouf 2023 [9]	0	0
Patra 2023 [29]	NR	NR
Anand 2022 [31]	NR	NR
Kastan 2022 [32]	NR	NR
Kiluba 2022 [30]	0	0
Kostek 2021 [33]	5	NR
Gujrathi 2021 [34]	0	0
Thomas 2020 [36]	NR	NR
Niranjan 2020 [37]	NR	NR
Islam 2020 [35]	0	0
Hemmaouil 2019 [10]	0	0
Saldanha 2019 [38]	0	0
Sreejayan 2019 [39]	1	Right
Sheikh 2019 [40]	NR	NR
Wojtczak 2018 [43]	1	Right
Shamkuwar 2016 [41]	2	NR
Saknure 2016 [42]	0	0
Nyeki 2015 [44]	0	0
Zada 2014 [45]	NR	NR
Idris 2013 [46]	0	0
Pradeep 2012 [47]	1	Right
Ashgharpour 2012 [48]	1	Right
Tang 2012 [49]	2	Right
Kaisha 2011 [50]	1	Right
Sunanda 2010 [51]	1	Right
Lee 2009 [52]	0	0
Makay 2008 [53]	0	0
Uen 2006 [54]	0	0
Ardito 2004 [55]	5	Right
Page 2003 [56]	0	0
Sun 2001 [57]	1	Right
Poyraz and Calguner 2001 [58]	0	0
Campos and Henrique 2000 [59]	0	0
Sturniolo et al. 1999 [60]	0	0
Salama and McGrath 1992 [61]	NR	NR
Lekacos et al. 1992 [62]	1	Right
Al-Salihi and Dabbagh 1989 [63]	0	0
Steinberg et al. 1986 [64]	NR	NR
Papadatos 1978 [65]	2	Right
Skandalakis et al. 1976 [66]	1	Right
Kratz 1973 [67]	NR	NR

## Discussion

This systematic review, integrating 60 studies published between 1973 and 2025 and encompassing more than 14,800 hemisomas, provides an updated and comprehensive synthesis of the topographic variability of the recurrent laryngeal nerve (RLN) in relation to the inferior thyroid artery (ITA) and its implications for thyroid surgery. The most consistent finding across the literature is the predominance of a posterior RLN course relative to the ITA [3]. This pattern, observed in 7,712 of 14,169 classified hemisomas, represents a robust signal spanning both classical anatomical dissections and contemporary intraoperative series, and offers a reliable empirical reference for surgical planning and dissection strategy [3, 47, 53]. Nevertheless, prevalence alone does not fully capture the clinical complexity highlighted by the available evidence. The anatomical landscape is markedly heterogeneous, with a substantial proportion of anterior and interposed (“between”) relationships that require continuous, adaptive interpretation during surgery [3, 47]. 

The presence of approximately 27% anterior courses and nearly 18% interposed courses indicates that so-called “non-standard” configurations are sufficiently common to challenge procedural automatisms [3]. In particular, when the RLN courses between ITA branches, dissection guided primarily by vascular landmarks may increase the risk of traction, compression, or thermal injury [3, 43]. In this context, assuming a stereotyped nerve–artery relationship before direct confirmation may lead to hazardous maneuvers. These findings can inform intraoperative orientation and may support a cautious, stepwise approach: careful topographic identification of the nerve, verification of the RLN–ITA relationship prior to arterial ligation, and selective use of neuromonitoring when anatomy appears atypical or exposure is compromised [1, 2, 43]. In parallel, the timing of ITA branch ligation should be adapted to the observed anatomy. Early, controlled ligation may facilitate exposure in anterior courses, whereas in interposed configurations, it is preferable to isolate and delineate the nerve before attempting vascular separation [3, 43]. Side-specific analyses further reveal a clinically relevant asymmetry. The left side demonstrates a more consistent predominance of the posterior RLN course, whereas the right side exhibits greater variability in RLN–ITA relationships [3, 47]. This asymmetry has practical implications. On the left, a posteromedial exploration may be a reasonable starting orientation in many cases; however, variants remain common and direct identification should guide the operative sequence [3]. On the right, a more flexible and cautious strategy is warranted, with a lower threshold to suspect associated vascular variants, including the non-recurrent laryngeal nerve (NRLN) [6, 7]. The identification of 39 NRLNs, all right-sided, underscores a rare but high-stakes scenario: failure to anticipate an NRLN can rapidly transform a routine procedure into a high-risk dissection when expected landmarks are absent [6, 7]. At the same time, the rarity of this variant does not justify indiscriminate use of adjunctive technologies. Instead, the pragmatic message is to integrate clinical judgment with confirmatory tools when vascular anomalies are suspected or when dissection does not reproduce anticipated topographic cues [2, 6]. 

Methodological appraisal provides essential context for the very high statistical heterogeneity observed across outcomes. AQUA-based assessment indicated overall moderate study quality. More recent publications generally report more explicit aims and more detailed methods, yet limitations remain common, particularly with respect to sampling design, systematic side-specific documentation, and transparency in reporting. [67] Importantly, classification and reporting issues extend beyond numerical considerations. Across the aggregated dataset, 651 hemisomas were reported as identified, but not classifiable with respect to the ITA relationship, and 249 RLNs were reported as not identified. Importantly, these missing/uncertain classifications are unlikely to be random: intraoperative series may under-classify or fail to identify the RLN in complex operative fields, whereas cadaveric dissections may differ in exposure and definition of ITA branching. This differential misclassification may bias pooled proportions in unpredictable directions, and therefore pooled estimates should be interpreted as orientation-level benchmarks.

These discrepancies likely reflect intrinsic differences between intraoperative settings—where bleeding, adhesions, and gland enlargement may obscure anatomy and cadaveric dissections, in which tissue planes are often better preserved but may be affected by postmortem artifacts [3, 47]. 

Our pooled proportions broadly align with prior syntheses on RLN–ITA topography, which consistently reported a predominance of a posterior course while emphasizing substantial variability and clinically relevant non-posterior patterns. Differences across reviews are plausibly explained by heterogeneous definitions (particularly for ‘interposed/between’ configurations and ITA branching), mixed inclusion of intraoperative versus cadaveric series, and temporal changes in surgical exposure and reporting standards. Importantly, our updated dataset reinforces that reliance on a single stereotyped landmark is unsafe, supporting a standardized reporting framework for RLN–ITA relationships [3]. 

Additional contributors include geographic and temporal variability, evolving documentation practices, inconsistent adoption of neuromonitoring, and heterogeneous operational definitions of anatomical categories. [2, 3, 67] Consequently, pooled estimates should be interpreted as clinically useful, orientation-level benchmarks rather than immutable truths: they provide a pragmatic anatomical map.Still, they cannot replace case-specific assessment by the operating surgeon [3]. 

The findings of this review also highlight clear methodological priorities for future research. Greater standardization is essential, including unambiguous operational definitions for anterior, posterior, and interposed categories; mandatory side-specific denominators; detailed reporting of neuromonitoring use and surgical technique; and consecutive or explicitly described sampling to reduce selection bias. [67] Multicenter studies adopting shared protocols would help mitigate geographic and technical fragmentation and yield more precise, generalizable estimates. From a clinical and educational perspective, training curricula should incorporate these data by emphasizing not only the “standard” anatomical pattern but also the recognition and management of variants, interpretation of vascular cues suggestive of atypical nerve courses, and appropriate use of neuromonitoring as a diagnostic adjunct rather than a substitute for meticulous dissection. [1–3, 67]

Across the reviewed evidence, a practical intraoperative principle consistently emerges: the sequence of surgical steps and the timing of arterial branch ligation should be modulated according to confirmed nerve topography. In challenging scenarios, deliberate pacing, prioritizing nerve isolation before ligation, and targeted neuromonitoring when anatomy is unclear are complementary strategies to reduce the risk of RLN injury [1, 2, 43]. Although the current evidence base does not yet support rigid prescriptive algorithms, it provides coherent directional guidance: favor posteromedial exploration when feasible, maintain heightened vigilance for variants on the right side, verify anatomy before ligation, and pursue greater standardization to strengthen future evidence. [2, 3, 67]

## Conclusions

This systematic review confirms that a posterior course of the recurrent laryngeal nerve relative to the inferior thyroid artery represents the most frequent anatomical configuration [3]. Additionally, the substantial prevalence of anterior and interposed variants underscores the need for caution and adaptability during thyroid surgery. A stepwise approach—based on direct visual identification, confirmation of the RLN–ITA relationship before arterial branch ligation, and selective use of neuromonitoring when exposure is limited—may be considered consistent with strategies aimed at minimizing RLN injury. Furthermore, greater methodological standardization in future anatomical studies is essential to reduce residual heterogeneity and to facilitate more reliable translation of anatomical evidence into shared operative strategies and structured surgical training pathways.

## Limitations

This study has several limitations. Temporal bias: included studies span 1973–2025, a period characterized by major changes in surgical exposure, documentation standards, which may affect anatomical reporting. Differential misclassification: cadaveric dissections and intraoperative series differ in exposure conditions and definitions and a relevant proportion of hemisomas were reported as identified but not classifiable (*n* = 651) or not identified (*n* = 249), potentially introducing non-random misclassification. Non-independence: some datasets may include bilateral sides from the same subject without available clustering information, which may affect precision of pooled estimates. Formal inference on left–right differences was not performed because side-level observations may not be independent and individual-level clustering information was unavailable in most datasets. Publication bias: we did not formally assess publication bias as these approaches are not well validated for extreme-heterogeneity proportion meta-analyses; therefore, selective reporting cannot be excluded.

## Data Availability

All data used in this study were extracted from published literature and are publicly available;

## Declarations

### **Registration and protocol**

This systematic review was registered with PROSPERO (CRD420251232692). The full protocol is available at https://www.crd.york.ac.uk/PROSPERO/view/CRD420251232692, and no amendments were made after registration.
